# A Visit

**Published:** 1870-02

**Authors:** 


### A VISIT.

During the late visit of Dr. Corydon Palmer to the Ohio College of Dental Surgery, he extended to us an invitation to visit him at his home, in Warren, Ohio, we, of course, made no delay in accepting.

We gave him time to get home, and, as soon thereafter as possible, we gathered our scanty together, and took a train on the Atlantic & Erie Railroad, at 9:45 P. M., and arrived on the outskirts of W. at 9:15, next A. M., after a blissful repose through the night, on the shelf of one of those dilectable public roosting places called a *palace* sleeping car.

Took a '*bus*; informed the driver where we wished to go, and was soon landed in front of "Elm Place."

The Doctor's office is built in cottage style—four rooms, with a veranda in front, latticed and beautifully adorned with running vines, giving a very cosy effect, and separate from his dwelling. This office has been occupied by Dr. P. for twenty-five or thirty years.

On stepping inside, we found a model suit of rooms, well arranged; his operating light is so arranged that he can control it, in quantity and direction. His laboratory is nearly all glass.

This information was obtained before the Doctor came in. By the time we had finished our glance around, he made his appearance, and received us cordially.

We could not have happened in at a more auspicious time, as it has always been our desire to witness the Doctor operating at his own chair; he was about to commence a series of operations, that very few would have selected, to exhibit his style upon, (we have seen big guns display upon pinhead cavities in the crown of superior first molars, at the National Association too), some of them being approximal cavities in the superior incisors, the teeth almost growing into one another, and very firmly set in the alveolus. We had seen rapid wedging before, but none so masterly as this; for, after placing the wedges in position, a few taps from his lead mallet forced the teeth apart, giving space enough to operate with ease, and such uninterrupted view as to detect the slightest imperfection in the operation.

In the preparation of cavities, Dr. P. always strikes for the weak points, never allowing frail walls to stand as monuments to inferior skill, or defective edges to remain; every part must be well defined, and the strongest part of the tooth made to sustain the filling.

To understand his method of preparing cavities one must know the instruments he uses. They will indicate for themselves the purposes for which they are designed. When taken in the hand, it seems as if they were almost intelligent, and required no will behind them.

For the introduction of gold, the Doctor has a system of pluggers. Each one has its part to perform in the operation, when that is finished, it is laid aside. Pluggers designed for one class of cavities are never used in any other, it is never necessary, because his designs and adaptations for each case are perfect.

Every operation is a study. The uniform excellence of the Doctor's operations may be attributed, in a great measure, to his systematic method—never using a plugger out of its place, or two or three different preparations of gold. If he commences a filling with foil, and he always does, he finishes with foil.

His instruments are comparatively few, all made for a definite use, and arranged upon a table within easy reach.

One finds no great, awkward-looking box, divided by partitions and drawers, called a dental case, and which, if judiciously arranged, might answer for a tenement house, or home for the friendless, with seventy or eighty pounds of useless ivory and steel scattered in disorder about them.

We have seen some cases which, if filled with Dr. P.'s system of instruments, would last the profession in any one of the States half a life time, if they were used properly.

Although Dr. P.'s skill as a mechanical Dentist does not fall below that of his operative, still he does very little in the way of dental mechanism. His laboratory is a model of neatness and order—a place for every thing and every thing in its place.

One cold winter day he was trying to find a place for his vulcanizer, so he took it down to the Mahoning river, cut a hole in the ice, and placed it in the river bed, together with the rest of his implements for doing rubber work. I ask pardon for giving old Josiah a clue to the whereabouts of these tools; but he will have to find the hole before he gets them.

The Doctor showed some models of artificial work, giving his plan of restoring the contour of face, lost by extracting; also some temporary teeth which he had mounted on plaster models, having saved them as they were shed from the mouths of his children. These models he used in explaining to parents points in regard to the first teeth.

He has another set of four pair of models, which were exhibited to the American Dental Association last summer. The first showing the correct arrangement of the natural teeth in a perfectly formed mouth ; the second showing the indications for filling; the third giving the improper methods of forming cavities; and the fourth, the proper manner of forming cavities. The models are all superior as works of art.

All of his instruments the Doctor makes himself, from the forging of the steel to the burnishing. The finest and best set of Dental instruments we know of are in his possession—his own handiwork.

During the last day of our visit, the Doctor took us to call upon some of his patients, to see more of his work. They all seemed to feel quite proud of their teeth, and willingly allowed an examination. In all his work is observable the close student of nature and lover of art, as every detail in ridge, cusp or depression is carefully and truthfully modeled.

We had the pleasure of examining the mouth of a beautiful little girl, in which the Doctor considers he has performed some of his finest operations ; the models he has in his possession showing the condition both before and after the mouth came under his care.

It is a delicate matter to take the Doctor to task for misrepresentation ; but the models fall so far short of the beauty of the work, that we feel it our duty. We will not venture a description; none could do it justice. The little patient deserves a great deal of credit for her courage and endurance, and she is intelligent enough to appreciate the efforts of her benefactor, more than can be said of most dental patients. All through the cases shown, the fillings were uniformly excellent, though a great many were made years ago, which the Doctor calls old fashioned work. The profession would have a higher standing among the peeple if more of such old fashioned work was done in these days.

We saw several cases of artificial work, made with reference to restoration of the features. Of course they were all successes, as any one who knows the man will know.

The Doctor has a new office nearly completed. Its arrangement is superb, appropriate, abundance of room, with economy of space, and appointments complete. May he have long years of happiness in his new quarters. We can not close without acknowledging the handsome treatment received at the hands of the Doctor's accomplished daughters. They are of that noblest class of young ladies who are thoroughly posted in culinary and household duties, as well as the higher branches of education, and are not affected with that abominable sickly sentimentality that renders the ability to make homes happy a vulgarity.

The brief time we spent at "Elm Place" was certainly the most pleasant and profitable we have ever spent anywhere. If any doubt our experience, and are so fortunate as to obtain an invitation, let them go and see.

January 24th, 1870. W. T.

